# The Effects of Patient Health Information Seeking in Online Health Communities on Patient Compliance in China: Social Perspective

**DOI:** 10.2196/38848

**Published:** 2023-01-09

**Authors:** Xinyi Lu

**Affiliations:** 1 School of Management and E-business Zhejiang Gongshang University Hangzhou China

**Keywords:** online health communities, OHCs, health information seeking, social presence, social support, perceived responsiveness

## Abstract

**Background:**

Online health communities (OHCs) can alleviate the uneven distribution and use of medical resources and severe hospital congestion. Patients may seek health information through OHCs before or after visiting physicians, which may affect their cognition, health literacy, decision-making preferences, and health-related behaviors such as compliance. Social factors (social support, social presence, and responsiveness) are closely related to patients’ health information–seeking behavior and are significantly considered in OHCs.

**Objective:**

This study aimed to explore the effects of patients’ health information–seeking behavior (way and effectiveness) on compliance with physicians from the perspectives of patients’ perceived social support, social presence, and responsiveness.

**Methods:**

This study established a research model from the perspective of social information processing by using the social exchange theory. An anonymous questionnaire survey was conducted with several Chinese OHCs to collect data. Partial least squares and structural equation modeling were adopted to test the hypotheses and develop the model.

**Results:**

This study received 403 responses, of which 332 were valid, giving a validity rate of 82.4% (332/403). Among the sample, 78.6% (261/332) of the individuals were aged between 20 and 40 years, 59.3% (197/332) were woman, 69.9% (232/332) lived in urban areas, and 50% (166/332) had at least a bachelor’s degree. The reliability, convergent validity, and discriminant validity were acceptable. Both the way and effectiveness of patients seeking health information through OHCs have a positive impact on their compliance through the mediation of their perceived social support, social presence, and responsiveness from OHCs and other users, and patient compliance can be improved by guiding patient health information–seeking behavior in OHCs from a social perspective.

**Conclusions:**

This study proposes a research model to corroborate that patient health information–seeking behavior (way and effectiveness) in OHCs exerts positive effects on patient compliance with the treatment and physician’s advice and provides suggestions for patients, physicians, and OHC service providers in China to help guide patients’ health-related behaviors through OHCs to improve patient compliance, patient satisfaction, treatment efficiency, and health outcomes.

## Introduction

### Background

Online health communities (OHCs) provide a platform for the public to seek health information and health care, communicate with physicians, and interact with other users through posts and web-based messages, breaking the limitations of time and space [1]. There are multiple forms of OHCs in China, and they can be divided into the following three categories according to their functions: (1) For patients, to communicate with physicians to seek health care and health information, such as “haodaifu”; (2) For patients, to communicate with other patients, such as “tianmijiayuan”; and (3) For physicians, to communicate with other physicians, such as “dingxiangyuan.” Patients with simple and minor symptoms can consult physicians and also ask for help from physicians in other regions through OHCs instead of visiting health care institutions. Therefore, OHCs can help people maintain a healthy lifestyle and reduce the burden on the health care system and are beneficial for alleviating the situation in which the distribution of medical resources is uneven, use is low, and hospital congestion is serious in China [2]. For example, during the COVID-19 outbreak, OHCs took advantage of the telemedicine function [3] to provide medical services and health information for Chinese people under mandatory home or centralized social isolation [4].

For patient rehabilitation and health maintenance, following medical prescriptions and maintaining a healthy lifestyle are critical [5]. Without face-to-face meetings and physician monitoring, self-management plays an important role in rehabilitation. Patient compliance is a critical factor that influences the effectiveness of self-management, especially for chronic diseases [6]. Haynes et al [7] defined patient compliance as “the extent to which a person’s behavior (in terms of taking medications, following diets or executing lifestyle changes) coincides with medical or health advice.” Tustin [8] corroborated that treatment regimens can be effective only if patients are highly compliant with the physicians’ advice. Therefore, people who comply with treatment are healthier than their peers [9,10]. Khera et al [11] showed that patient compliance is conducive to the prevention of cardiovascular diseases.

Patient compliance has significant effects on the economy and society [12]. For example, physicians advise the public to isolate themselves at home during the COVID-19 outbreak, but some people do not comply and insist on going out without wearing a mask. Such noncompliance may cause the spread of COVID-19 and social panic, leading to economic losses. Fortunately, patient compliance can be improved through guidance and intervention because this parameter is dynamic [13]. In general, most patients may not intentionally refuse to comply with treatments and physicians’ advice but do not comply because of cognitive deficiency [13]. At times, psychosocial stress may cause individuals to have insufficient energy to conduct compliant activities.

Web-based health information can be divided into the following two categories [14-16]: (1) medical-related information, which can help patients choose suitable hospitals and physicians and cooperate with physicians to make decisions and address medical issues and (2) lifestyle-related information, such as maintaining a balanced diet, controlling weight, and exercising, which can help patients maintain a healthy lifestyle in daily life. Individual information behaviors have been extended to the context of the internet. As Wilson [17] defined, individual health information behaviors in OHCs include health information seeking, health information use, communication, and passive receiving health information behaviors. Patients are likely to seek health information before or after visiting physicians [18], which may substantially affect their cognition, health literacy, decision-making preferences, and health-related behaviors [19,20] such as patient compliance. For example, patients may be unwilling to comply with their physicians’ advice if they find that the health information from physicians is inconsistent with that obtained by themselves. Therefore, this study intends to explore ways to improve patient compliance by guiding patient health information–seeking behavior through OHCs.

In the OHCs discussed in this study, patients engage in social activities with other users (physicians and patients) [21]. Social factors are closely related to patient health-related behaviors, health outcomes, and adherence [22,23]. The perception of social presence enables patients to continuously use OHCs, trust other users [24], and gain positive emotions [25]. In addition, web-based social presence has a positive effect on offline health behaviors [26]. Perceived responsiveness encourages patients to repeatedly seek health information and share experiences through OHCs and also enhances their self-efficacy [27], thereby improving their health literacy and ability to manage themselves. The physician-patient relationship is an important social relationship in OHCs [28] and can exert a significant effect on patient compliance [29]. Through social interactions, patients can share their experiences and knowledge with other users and subsequently obtain health information in return [30].

Considerable research has focused on OHCs from a social perspective, but few have explored the effect of health information–seeking behavior on patient compliance from the mediating perspective of social factors. Moreover, this study was conducted in China, where health care system development differs from that in other countries and regions. Therefore, this study aims to identify the effect of patients’ health information–seeking behavior in OHCs on their compliance, considering the mediating effects of patients’ perceived social support, social presence, and responsiveness.

### Research Model and Hypotheses

#### Perceived Social Support

Social support is defined as resource exchange between at least 2 individuals—the provider and the recipient—to improve the latter’s well-being [31,32]. Social support can be divided into 4 categories, namely, informational support, emotional support, companionship support, and instrumental support [32-34]. In OHCs, the main forms of social support are informational and emotional support [35,36], which can be exchanged through social interactions among members [30]. Perceived social support is closely related to patients’ health-related behaviors and health outcomes [22,37]. When patients are aware that they have received social support from other OHC members, they will believe that they have received care, love, and respect from their friends [38]. Thus, their perceived risk can be reduced, and their trust is given to people who provide social support [39].

#### Perceived Social Presence

Social presence is defined as “an emotional experience that arises from the communication process, including a feeling of closeness or a sense of belonging to a relationship, a community, or a group” [40,41]. Face-to-face communication or meetings are not necessary for people to perceive social presence or develop or maintain relationships [41]. Therefore, members are likely to perceive social presence in OHCs, which may enhance the feelings and usefulness of the communities [26]. Effective information seeking and communication require patients’ attention, trust, and willingness [42]. When patients perceive social presence in interactions through OHCs, they are more likely to ask for help and support and to trust other members [26]. Perceiving social presence has a positive effect on users’ continued use of web-based platforms [43] and can encourage patients to participate in OHCs’ social activities, which are beneficial for developing their sociability [26]. Moreover, perceived social presence in OHCs may influence patients’ offline health-related behaviors such as maintaining a balanced diet [44].

#### Perceived Responsiveness

In OHCs, perceived responsiveness refers to a user’s perception that other members are willing to interact and provide help [27,45]. Users who receive responses after publishing posts regard OHCs as a useful tool for conducting health-related activities and are more likely to reuse the platforms [46]. In addition, perceiving high responsiveness may prompt users to introduce OHCs to their relatives and friends, thereby attracting new members [46]. By contrast, users may feel disappointed and refuse to use OHCs if their posts or queries do not receive any response [27]. Moreover, people who perceive high responsiveness from OHCs feel that they have received reciprocal benefits from others [47] and in return sincerely share their health-related experiences and information with other members. Accordingly, people who use OHCs to seek health information can effectively use the information and improve their health literacy with the help of other members.

#### Research Hypotheses

As an active behavior, health information–seeking behavior in OHCs requires patient willingness and health literacy. Otherwise, patients may be unable to obtain suitable or reliable health information and may even trust low-quality or incorrect health information, which may adversely affect their health outcomes. Moreover, OHCs are characterized by zero gatekeeping and cost publishing [48]. In addition to the patient’s ability to seek health information through OHCs, the effectiveness of such behavior plays an important role in their daily lives and the health care system [49]. Effectiveness refers to the success of patients receiving health information from OHCs [50]. Therefore, this study examines patient health information–seeking behavior in OHCs with 2 dimensions—way and effectiveness.

From a social information processing perspective [20], individuals seek information from other sources if they do not grasp the information they need. This information can affect their attitudes, beliefs, and opinions and ultimately influence their behaviors [51,52]. In this study, patients sought health information through OHCs, which may influence their views and health behaviors. Resource exchange occurred between physicians and patients, indicating a social exchange relationship [5,53,54]. Resources that are likely to be exchanged between physicians and patients include therapies, prescriptions, trust, satisfaction, and empathy. Health information obtained from OHCs can influence patients’ perceptions and feelings about OHCs, the health care system, physicians, and the physician-patient relationship. In this case, according to the social exchange theory [55], the patient resource exchange behavior in the physician-patient relationship may change. For example, patients may compare the health information obtained from OHCs with that obtained from physicians and thereby consider their physicians reliable if the 2 are consistent. Hence, patients are encouraged to trust their physicians and comply with their advice.

**Figure 1:**
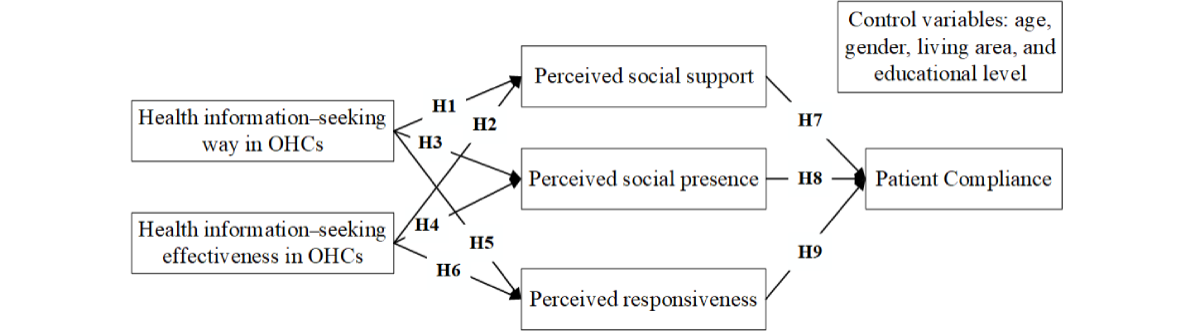
Research model. OHC: Online health communities.

Under the guidance of the social exchange theory, this study established a research model (Figure 1) from a social information processing perspective. Previous studies have shown that seeking health information through OHCs allows patients to perceive social support from other members [56,57]. However, not all patients perceive social support through health information–seeking behaviors. Patients who cannot seek health information in OHCs in suitable ways are less likely to receive responses than those who understand the various ways of information seeking. For example, patients may be unable to interact with other OHC users if they do not know how to publish posts, reply, or communicate through web-based messages. In addition, patients who seek health information through OHCs with low effectiveness may not consider the platform to be useful, which decreases their perceived social support. Therefore, we proposed the following hypotheses:

- Hypothesis H1: The way patients seek health information in OHCs has a positive effect on their perceived social support.
- Hypothesis H2: The effectiveness of patients seeking health information in OHCs has a positive effect on their perceived social support.

Seeking health information in various suitable ways in OHCs can provide patients with feelings of warmth and sociability. First, patients who seek health information in various ways are more likely to participate in social interactions with other OHC users and perceive social presence than those who adopt a single or incorrect method. Second, patients can increase their familiarity with OHCs regarding procedures and functions if they seek health information in different ways [24]. Therefore, we proposed the following hypothesis:

- Hypothesis H3: The way patients seek health information in OHCs has a positive effect on their perceived social presence.

Health information seeking is highly effective when patients effectively browse OHCs and communicate with others through posts and web-based messages. In addition, seeking health information with high effectiveness makes patients perceive that OHCs are useful and helpful and that users are warm hearted. Thus, patients can have emotional experiences through social interactions and perceive that OHCs are easy to use, welcoming, and socially friendly, which can increase their perceived social presence. Accordingly, we proposed the following hypothesis:

- Hypothesis H4: The effectiveness of patients seeking health information in OHCs has a positive effect on their perceived social presence.

From a social information processing perspective, seeking health information has the potential to change patients’ mental states and attitudes [51,52]. In other words, health information–seeking behavior can make patients perceive responsiveness to OHCs’ specific situations. Seeking health information in various ways can improve the possibility of patients interacting with other members of OHCs, ultimately increasing the possibility of perceiving responsiveness. In addition, the high effectiveness of health information seeking means that reliable, readily usable, and relevant information has been obtained [58,59], which makes patients receive high responsiveness from OHCs and other members. The above discussion leads to the following hypotheses:

- Hypothesis H5: The way patients seek health information in OHCs has a positive effect on their perceived responsiveness.
- Hypothesis H6: The effectiveness of patients seeking health information in OHCs has a positive effect on their perceived responsiveness.

Hanson et al [60] corroborated that social competence is beneficial for improving patient compliance. Informational and emotional support from OHCs can reduce the perceived risk [39] and improve patient health literacy and self-efficacy to a certain extent [36,61]. Patients who perceive social presence can notice self-control, which enables their self-confidence to solve health problems [62]. Social presence in OHCs also affects offline health behaviors [26,44]. High perceived responsiveness enables patients to realize that they have obtained useful health information from which they can benefit, and then patients will participate further in social interactions with other members of OHCs [27]. Therefore, patients are encouraged to participate in communication with their physicians [28], and their ability to discuss health-related issues with others can be improved. In the offline treatment process, patients discuss and compare the information they obtain from OHCs with that of physicians. Patients learn much about the treatment options, costs, risks, and benefits from physicians. This deep physician-patient communication can demonstrate the professionalism and empathy of physicians. According to the social exchange theory, patients give their trust and compliance to physicians as a resource exchange [53,54]. This situation led us to derive the following hypotheses:

- Hypothesis H7: Perceived social support in OHCs has a positive effect on patient compliance.
- Hypothesis H8: Perceived social presence in OHCs has a positive effect on patient compliance.
- Hypothesis H9: Perceived responsiveness in OHCs has a positive effect on patient compliance.

## Methods

### Instrument Development

This study used multiple-item scales validated in previous studies to measure constructs covering the research variables. As shown in Multimedia Appendix 1, the survey items were measured using a 7-point Likert-type response format (strongly disagree=1, strongly agree=7). Specifically, the way patients seek health information in OHCs refers to “the way people search for and apply both active and passive information,” which was measured using a 4-item scale from Khazaee-Pool et al [63]. The effectiveness of health information–seeking in OHCs was measured using a 5-item scale adopted from Nambisan [49] combined with 2 previous studies [58,59]. Nambisan [49] likewise adapted a 5-item scale from Zimet et al [64] to measure social support. Perceived social presence was measured using a 5-item scale developed by Gefen and Detmar [65]. Perceived responsiveness was measured using a 3-item scale developed by Ridings et al [47]. A 5-item scale adopted by Laugesen et al [66] from Hausman [67] was used to measure patient compliance.

Before the investigation, we translated the English questionnaire into Chinese and considered the cross-cultural adaptation. In addition, several people with experience seeking health information through OHCs were invited to fill out the questionnaire and provide advice for revising the instrument in Chinese. Finally, we back-translated the Chinese questionnaire into English to evaluate whether the concepts between the 2 versions were consistent [68-70].

Considering the possible effects of demographic variables on patient health information–seeking behavior and other health-related behaviors, this study regarded age, gender, living area, and education level as control variables in the analysis. Age was self-reported by year. Gender (man=1, woman=2); living area (urban=1, rural=2); and education level (junior middle school or below=1, high school=2, junior college=3, bachelor’s degree=4, master’s degree=5, and PhD=6) were dummy coded.

### Data Collection and Respondent Profile

The investigation was conducted anonymously in several Chinese OHCs in March 2020. The participants were Chinese people who had sought health information through OHCs within the previous month such that they could recall their relevant experiences and feelings. Of the 403 responses received, 332 were valid, with a validity rate of 82.4% (332/403). Table 1 shows that among the sample, 78.6% (261/332) were aged between 20 and 40 years, 59.3% (197/332) were woman, 69.9% (232/332) lived in urban areas, and 50% (166/332) had at least a bachelor’s degree. The demographics of our sample were consistent with those of OHC users characterized as young, woman, living in urban regions, and highly educated [28,71,72]. Thus, the sample can be used to analyze the research model.

**Table 1:** Sample demographics (N=332).

Demographic characteristics			Values, n (%)
**Age (years)**			
	<20	5 (1.5)	
	20-29	100 (30.1)	
	30-39	161 (48.5)	
	40-49	54 (16.3)	
	50-59	11 (3.3)	
	≥60	1 (0.3)	
**Gender**			
	Man	135 (40.7)	
	Woman	197 (59.3)	
**Living area**			
	Urban	232 (69.9)	
	Rural	100 (30.1)	
**Educational level**			
	Junior middle school or below	5 (1.5)	
	High school	35 (10.5)	
	Junior college	126 (38)	
	Bachelor’s degree	104 (31.3)	
	Master’s degree	39 (11.7)	
	PhD	23 (6.9)	

### Reliability and Validity Evaluation

Data analysis was conducted using the SPSS software (version 22.0; IBM Corp) and SmartPLS software (version 3.2.8; SmartPLS GmbH). Despite validation in previous studies, the scales were re-evaluated for reliability and validity because the specific background and participants of this study differ from those of previous studies. The Cronbach α was used to assess the reliability of scales. Table 2 presents the results, where each value was greater than the threshold of .700, indicating acceptable reliability [73]. Confirmatory factor analysis was used to analyze convergent and discriminant validity. The Kaiser-Meyer-Olkin value was .961, so the data could be used for factor analysis [74].

Tables 3 and 4 show the validity results, including composite reliability, average variance extracted (AVE), and correlations between constructs. All composite reliability values were greater than the cutoff value of .700, and the AVEs were greater than the cutoff value of .500. Therefore, convergent validity was acceptable [75]. The square root of the AVE of each construct exceeded the correlation coefficients between this construct and the other constructs [75]. Therefore, the discriminant validity of the instrument used in this study was acceptable.

**Table 2:** Cronbach α of constructs.

Construct	Cronbach α
Way of health information seeking in OHC^a^	.738
Effectiveness of health information seeking in OHCs	.860
Perceived social support	.859
Perceived social presence	.867
Perceived responsiveness	.726
Patient compliance	.843

^a^OHC: online health community.

**Table 3:** Composite reliability and average variance extracted.

Construct	CR^a^	AVE^b^	Sqrt^c^ AVE
Way of health information seeking in OHCs^d^	0.836	0.560	0.748
Effectiveness of health information seeking in OHCs	0.900	0.642	0.801
Perceived social support	0.899	0.640	0.800
Perceived social presence	0.904	0.653	0.808
Perceived responsiveness	0.845	0.646	0.804
Patient compliance	0.889	0.616	0.785

^a^CR: composite reliability.

^b^AVE: average variance extracted.

^c^Sqrt: square root.

^d^OHC: online health community.

**Table 4:** Correlations between constructs.

Construct	WHIS^a^	EHIS^b^	PSS^c^	PSP^d^	PR^e^	PC^f^
WHIS	0.748	—^g^	—	—	—	—
EHIS	0.735	0.801	—	—	—	—
PSS	0.730	0.759	0.800	—	—	—
PSP	0.728	0.697	0.771	0.808	—	—
PR	0.687	0.709	0.752	0.797	0.804	—
PC	0.733	0.694	0.775	0.769	0.736	0.785

^a^WHIS: way of health information seeking in online health communities.

^b^EHIS: effectiveness of health information seeking in online health communities.

^c^PSS: perceived social support.

^d^PSP: perceived social presence.

^e^PR: perceived responsiveness.

^f^PC: patient compliance.

^g^Not applicable.

## Results

First, we analyzed the effects of the control variables (demographic factors) on the research model. Gender had a positive effect on perceived responsiveness, whereas living areas had a positive effect on perceived social presence. Specifically, female patients were more likely to perceive responsiveness in OHCs than male patients, and those who lived in urban areas perceived higher social presence than those who lived in rural areas. Furthermore, we analyzed the effect size of control variables using Cohen *ƒ*^2^ [76] (insignificant: *ƒ*^2^<0.020; small: 0.020≤*ƒ*^2^<0.150; medium: 0.150≤*ƒ*^2^<0.300; and large: *ƒ*^2^≥0.350). Table 5 shows the results of the multivariate coefficient of determination (*R*^2^) and *ƒ*^2^, which indicate that the control variables only had a weak effect on perceived responsiveness with a small effect size.

As shown in Table 6, all the hypotheses were supported. In addition, we evaluated the effect sizes of the variables using Cohen *ƒ*^2^ (Table 7). The results showed that the way of health information seeking in OHCs had strong effects on patients’ perceived social support and perceived social presence with medium effect sizes but had a weak effect on patients’ perceived responsiveness with a small effect size. Regarding the effectiveness of seeking health information in OHCs, its effect sizes on patients’ perceived social support and responsiveness were medium, whereas its effect size on patients’ perceived social presence was small. Social presence and responsiveness perceived by patients had weak effects on patient compliance with small effect sizes. Meanwhile, perceived social support had a strong effect on patient compliance with a medium effect size. In addition, the path coefficients from the effectiveness of patients seeking health information to the 3 mediators (0.468, 0.477, and 0.423) are greater than those from the way patients seek health information to the 3 mediators (0.387, 0.369, and 0.362, respectively), indicating that the effectiveness of patient health information seeking had a stronger effect on patient compliance.

**Table 5:** Multivariate coefficient of determination (R2) results.

Variables			*R* ^2^				Δ*R*^2a^		*ƒ* ^2b^		Effect size
			In		Out						
**Perceived social support**											
	Control variables	0.646		0.640		0.006		0.017		Insignificant	
	Age	0.646		0.646		0		0.000		Insignificant	
	Gender	0.646		0.643		0.003		0.008		Insignificant	
	Living area	0.646		0.646		0		0.000		Insignificant	
	Educational level	0.646		0.644		0.002		0.006		Insignificant	
**Perceived social presence**											
	Control variables	0.594		0.587		0.007		0.017		Insignificant	
	Age	0.594		0.594		0		0.000		Insignificant	
	Gender	0.594		0.593		0.001		0.002		Insignificant	
	Living area	0.594		0.590		0.004		0.010		Insignificant	
	Educational level	0.594		0.592		0.002		0.005		Insignificant	
**Perceived responsiveness**											
	Control variables	0.577		0.562		0.015		0.035		Small	
	Age	0.577		0.572		0.005		0.012		Insignificant	
	Gender	0.577		0.569		0.008		0.019		Insignificant	
	Living area	0.577		0.576		0.001		0.002		Insignificant	
	Educational level	0.577		0.575		0.002		0.005		Insignificant	
**Patient compliance**											
	Control variables	0.687		0.684		0.003		0.010		Insignificant	
	Age	0.687		0.687		0		0.000		Insignificant	
	Gender	0.687		0.687		0		0.000		Insignificant	
	Living area	0.687		0.685		0.002		0.006		Insignificant	
	Educational level	0.687		0.686		0.001		0.003		Insignificant	

^a^*Δ*R^2^: *R*^2^_In_−*R*^2^_Out_.

^b^*ƒ*^2^: Cohen *ƒ*^2^.

**Table 6:** Results of hypothesis testing.

Hypothesis	Path coefficient	*t* test^a^ (*df*)	*P* value
H1: The way patients seek health information in OHCs^b^ has a positive effect on their perceived social support.	0.387	6.203 (315)	<.001
H2: The effectiveness of patients seeking health information in OHCs has a positive effect on their perceived social support.	0.468	7.035 (315)	<.001
H3: The way patients seek health information in OHCs has a positive effect on their perceived social presence.	0.369	5.260 (315)	<.001
H4: The effectiveness of patients seeking health information in OHCs has a positive effect on their perceived social presence.	0.477	7.655 (315)	<.001
H5: The way patients seek health information in OHCs has a positive effect on their perceived responsiveness.	0.362	4.459 (315)	<.001
H6: The effectiveness of patients seeking health information in OHCs has a positive effect on their perceived responsiveness.	0.423	6.026 (315)	<.001
H7: Perceived social support in OHCs has a positive effect on patient compliance.	0.383	5.741 (315)	<.001
H8: Perceived social presence in OHCs has a positive effect on patient compliance.	0.313	5.154 (315)	<.001
H9: Perceived responsiveness in OHCs has a positive effect on patient compliance.	0.203	3.441 (315)	<.001

^a^*t* test was 2-tailed.

^b^OHC: online health community.

**Table 7:** Partial least squares effect size analysis.

Variables			*R* ^2a^				ΔR^2b^		*ƒ* ^2c^		Effect size
			In		Out						
**Patient compliance**											
	Perceived social support	0.687		0.634		0.053		0.169		Medium	
	Perceived social presence	0.687		0.659		0.028		0.089		Small	
	Perceived responsiveness	0.687		0.675		0.012		0.038		Small	
**Perceived social support**											
	Way of health information seeking in OHCs^d^	0.646		0.579		0.067		0.189		Medium	
	Effectiveness of health information seeking in OHCs	0.646		0.547		0.099		0.280		Medium	
**Perceived social presence**											
	Way of health information seeking in OHCs	0.594		0.498		0.096		0.236		Medium	
	Effectiveness of health information seeking in OHCs	0.594		0.538		0.056		0.138		Small	
**Perceived responsiveness**											
	Way of health information seeking in OHCs	0.577		0.516		0.061		0.144		Small	
	Effectiveness of health information seeking in OHCs	0.577		0.499		0.078		0.184		Medium	

^a^*R*^2^: Multivariate coefficient of determination.

^b^ΔR^2^=R^2^_In_−R^2^_Out_.

^c^*ƒ*^2^: Cohen *ƒ*^2^.

^d^OHC: online health community.

## Discussion

### Principal Findings

In this study, we proved that the way and effectiveness of patients seeking health information in OHCs have positive effects on patient compliance, and patients’ perceived social support, social presence, and responsiveness from OHCs played mediating roles in the research model. This study presents theoretical contributions to future studies on patients’ web-based information behaviors, social factors of OHCs, and patient compliance. In addition, patient information seeking behavior in OHCs is investigated by using the social exchange theory together with the social information processing perspective, which enriches the application of the social exchange theory and the social information processing approach in the context of OHCs and the field of health care.

This study has several practical implications for improving patient compliance and guiding patient health information–seeking behaviors through OHCs. First, the effectiveness of patients seeking health information is the preferred perspective for improving patient compliance. As Nambisan’s [49] survey shows, the information-seeking effectiveness has an effect on patients’ perceived empathy in OHCs, and then Lu and Zhang [77] further proved that patients’ perceived empathy in OHCs has an effect on patient compliance. Therefore, the readability, reliability, relevance, and timeliness of the health information provided to OHC users require priority. For example, OHCs can strengthen the management and monitoring of information quality so that the effectiveness of seeking health information can be improved accordingly and make platforms easy and friendly to use to help patients seek information conveniently and quickly. In addition, physicians can help develop their patients’ ability to seek health information with high effectiveness.

Second, patient compliance can be improved by enriching the ways patients seek health information through OHCs. As summarized by Lu and Zhang [2], there are various ways for patients to seek health information through OHCs, such as communicating with physicians, interacting with other members (ie, posts and web-based private messages), and browsing consultation text and posts, which can help prompt patients’ social feelings. Jiang and Street [56], Li and Mao [78], and Resnick et al [46] proved that patients communicating with physicians through web-based platforms would perceive more social support, social presence, and responsiveness, respectively, supporting our findings. In addition, as diagnoses and therapies vary from person to person, simply browsing others’ posts and consultation text might not help patients seek suitable and reliable high-quality health information and may even cause misdiagnosis. For example, Guan et al [79] proved that the predictors of severe scrub typhus in pediatric patients are different from those in elderly patients and that therapies are also different. Hence, physicians can encourage their offline patients to seek health information through OHCs in various ways, and OHCs can provide detailed guidelines regarding different methods of seeking health information for users.

**Figure 2:**
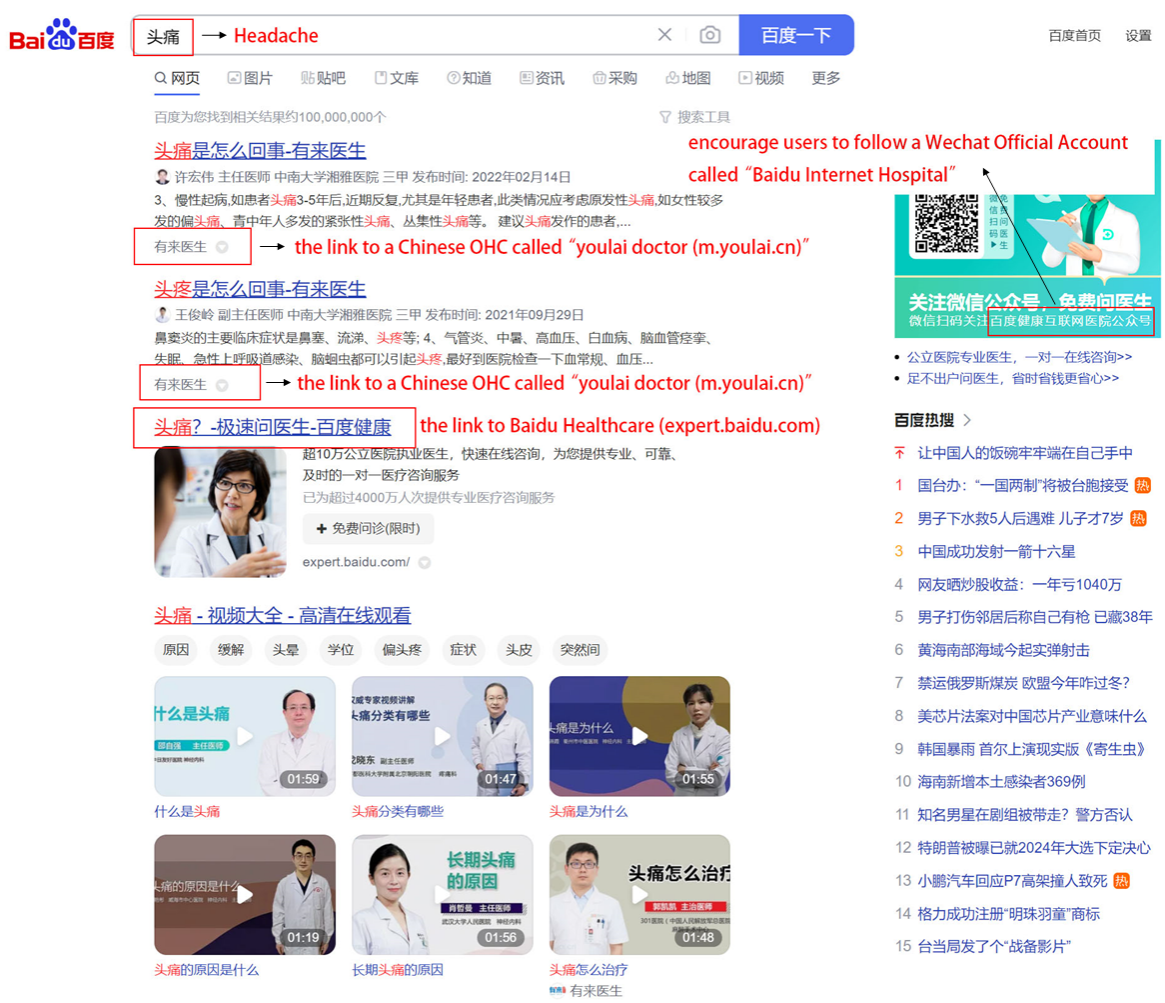
Top results of searching “headache” through Baidu search engine.

Patients using OHCs is a necessary prerequisite for improving effectiveness and enriching the ways of patient information–seeking behavior. Undoubtedly, patients do not have to use OHCs, especially those who do not have adequate eHealth literacy. Lu and Zhang [80] found that patients with inadequate eHealth literacy might be unable to achieve high effectiveness in seeking health information through OHCs. However, patients may inevitably contact OHCs because of the convenience of the internet, especially in China. To specify, on the one hand, physicians who practice in some Chinese OHCs, such as “haodaifu,” must pass the real-name and professional authentication. Patients can comment on physicians, send web-based gifts, and vote on physicians after web-based treatment, which constitutes physicians’ web-based word-of-mouth feedback. Accordingly, in offline treatment, physicians may encourage their patients to use OHCs in follow-up treatments to improve their web-based word of mouth. By contrast, Chinese people tend to seek information about their symptoms through search engines when they feel ill, and the top results of some Chinese search engines such as Baidu are often linked to OHCs (Figure 2). In such cases, patients are more likely to visit the OHC portals. Moreover, Chinese people who live in some regions may be less likely to seek offline health care services because of the uneven distribution of medical resources [81,82] and the serious hospital congestion, so they would like to ask for help through OHCs.

Therefore, patients with limited eHealth literacy may also need OHCs. In addition, as supported by the survey by Kim et al [83], some individuals with inadequate eHealth literacy might not be aware of their limited level of eHealth literacy. In other words, some patients have high self-efficacy in seeking health information but do not have sufficient ability to accurately judge information quality and describe their symptoms and feelings. Moreover, these patients may misunderstand physicians’ advice and believe in false advertisements, which can decrease the effectiveness of health information–seeking through OHCs. Accordingly, patients may need to improve their eHealth literacy.

Third, patient compliance can be improved by guiding patient health information–seeking behavior from the following 3 social factors: social support, social presence, and responsiveness; social support is the most important factor related to interpersonal interactions in current and developing OHCs. This finding is consistent with the study by Bronstein [35], who proposed that providing or seeking social support is an important motivation for individuals to participate in OHCs’ activities. Furthermore, according to Audrain-Pontevia and Menvielle [28] and Hajli et al [30], social support is beneficial for self-management and strengthens the physician-patient relationship. Therefore, physicians should provide patients with informational and emotional social support through OHCs to improve patient compliance.

Similarly, social presence and responsiveness are important factors influencing OHC users’ behaviors and can be considered to improve patient compliance in this study. Zhang et al [84] corroborated that increasing the frequency and quality of social interactions between physicians and patients and adopting various interaction methods (ie, text, image, and video) are useful for improving patients’ perceptions of social presence and responsiveness. This finding inspired us to propose that OHCs can develop several interaction tools to improve the social presence perceived by users, which was also supported by Hajli et al [24]. For example, OHCs can adopt some incentives such as providing reward points that can offset the cost of web-based consultation, encouraging users to create posts, sharing health information and interacting with others, and seeking health care services on the web to improve the sense of social presence.

In terms of responsiveness, Resnick et al [46] proposed that forums’ staff can respond when members have not received a timely response, which inspired us to suggest that OHCs can develop a recommendation algorithm to automatically recommend health information according to users’ information seeking histories and preferences. In that case, OHC users can receive social support and perceive responsiveness even though they cannot quickly receive responses from physicians or other members.

### Limitations and Future Research

This study has several limitations and prospects. First, in addition to social factors, other angles can be considered to explore how to improve patient compliance with treatment and physician’s advice by guiding patient health information–seeking behavior through OHCs in future studies. Second, this study paid attention to the way and effectiveness of patient health information–seeking behavior in a broad sense but ignored the types of this behavior. In fact, patients who engage in different types of health information–seeking behaviors may adopt different ways and achieve different levels of effectiveness. For example, some patients who prefer to seek health information related to self-management skills and lifestyles are likely to browse web-based posts and articles; some, meanwhile, would prefer to browse physicians’ homepages, patient comments, and question and answer blocks to obtain information related to hospitals, physicians, and therapies. Moreover, patients with different potential illnesses may focus on different topics. Therefore, in future studies, we can investigate web-based health information behavior in the context of specific diseases such as diabetes. In addition, the type of health information–seeking behavior can be included in the research model to examine its effect on patient compliance. Third, we tested the hypotheses by analyzing data collected from a cross-sectional survey in the context of China but did not perform external validation, so the generalizability of our research model should be further determined [85]. In addition, the research model needs to be examined outside of China, and the similarities and differences between other countries and regions and China should be explored. Fourth, this study statically investigated the association between patient health information–seeking behavior (way and effectiveness) in OHCs and patient compliance from social perspectives but did not capture the dynamic changes in participants’ behaviors and attitudes in the longitudinal section, and the temporal sequence was questionable [85].

### Conclusions

This study proposes a research model to corroborate that patient health information–seeking behavior (way and effectiveness) in OHCs exerts positive effects on patient compliance with the treatment and physician’s advice from the mediations of patients’ perceived social support, social presence, and responsiveness. This study provides suggestions for patients, physicians, and OHC service providers in China to help guide patient health-related behaviors through OHCs to improve patient compliance, patient satisfaction, treatment efficiency, and health outcomes. In the follow-up work, this model should undergo examinations with populations from other countries and regions to further assess its acceptability.

## Appendix

Multimedia Appendix 1 Survey questions.

## Abbreviations

AVE: average variance extracted
OHC: online health community
