# Supplementary material for: The Effects of Patient Health Information Seeking in Online Health Communities on Patient Compliance in China: Social Perspective
Source: J Med Internet Res. 2023 Jan 9;25:e38848. doi: 10.2196/38848 (PMC9871880; doi:10.2196/38848)
Supplement: Multimedia Appendix 1 [file jmir_v25i1e38848_app1.docx]

Survey questions

General information

1. Age:
2. Gender: ◻Male ◻Female
3. Living area: ◻Urban ◻Rural
4. Education level:

◻Junior middle school or below ◻High school ◻Junior college

◻Bachelor’s degree ◻Master’s degree ◻Ph.D.

1. What’s the last time you used online health communities (haodaifu, dingxiangyuan, chunyuyisheng, etc) to ask for physician’s help?

◻Within a week ◻Within a month ◻Within three months

◻More than half a year ◻More than a year ◻Never used

Measurement Instruments

(7-point Likert: strongly disagree, disagree, somewhat disagree, neither agree nor disagree, somewhat agree, agree, strongly agree)

1. Perceived responsiveness

(1) The people in OHCs are very responsive to my posts

(2) In OHCs, I can always count on getting adequate responses to my posts

(3) In OHCs, I can always count on getting responses to my posts fairly quickly

1. Perceived social presence

(1) There is a sense of human contact in OHCs

(2) There is a sense of personalness in OHCs

(3) There is a sense of sociability in OHCs

(4) There is a sense of human warmth in OHCs

(5) There is a sense of human sensitivity in OHCs

1. Perceived social support

(1) I could count on my friends in OHCs when things went wrong

(2) I had friends in OHCs with whom I could share my joys and sorrows

(3) I got the emotional help and support I needed from OHCs

(4) I could talk about my problems with the members in OHCs

(5) I knew someone in OHCs who was a real source of comfort to me

1. The effectiveness of health information seeking

(1) The health information that I obtained from OHCs is readily usable

(2) The health information that I obtained from OHCs is credible

(3) The health information that I obtained from OHCs is relevant

(4) The health information that I obtained from OHCs is reliable

(5) I obtained health information from OHCs in a timely manner

1. The way of health information seeking

(1) I am going to get new information and skills from OHCs to improve my health

(2) I can get follow-up on new programs related to healthcare from OHCs

(3) Having a suitable relationship with others in OHCs helps me to share information on illness prevention

(4) In OHCs, I talk to my health care provider about how to perform self-monitoring even if I have difficulty understanding him or her

1. Patient compliance

(1) I am following/did follow the suggestions of physician(s) from OHCs exactly

(2) I am following/did follow the drug/medication recommendations of physician(s) from OHCs

(3) I am following/did follow the orders of physician(s) from OHCs, such as to stay in bed

(4) I have returned or plan to return to the physician(s) from OHCs on the schedule suggested

(5) I have had or plan to have the follow-up tests recommended by the physician(s) from OHCs
